# Genome-wide placental DNA methylations in fetal overgrowth and associations with leptin, adiponectin and fetal growth factors

**DOI:** 10.1186/s13148-022-01412-6

**Published:** 2022-12-30

**Authors:** Meng-Nan Yang, Rong Huang, Tao Zheng, Yu Dong, Wen-Juan Wang, Ya-Jie Xu, Vrati Mehra, Guang-Di Zhou, Xin Liu, Hua He, Fang Fang, Fei Li, Jian-Gao Fan, Jun Zhang, Fengxiu Ouyang, Laurent Briollais, Jiong Li, Zhong-Cheng Luo, for the Shanghai Birth Cohort

**Affiliations:** 1 Ministry of Education-Shanghai Key Laboratory of Children’s Environmental Health, Early Life Health Institute, Department of Pediatrics, Xinhua Hospital, Shanghai Jiao-Tong University School of Medicine, Shanghai, 200092 China; 2 Lunenfeld-Tanenbaum Research Institute, Prosserman Centre for Population Health Research, Department of Obstetrics and Gynecology, Mount Sinai Hospital, Faculty of Medicine, University of Toronto, L5-240, Murray Street 60, Toronto, ON M5G 1X5 Canada; 3 Department of Obstetrics and Gynecology, Xinhua Hospital, Shanghai Jiao-Tong University School of Medicine, Shanghai, 200092 China; 4 Center for Fatty Liver, Shanghai Key Lab of Pediatric Gastroenterology and Nutrition, Department of Gastroenterology, Xinhua Hospital, Shanghai Jiao Tong University School of Medicine, Shanghai, 200092 China; 5 Department of Clinical Medicine-Department of Clinical Epidemiology, Aarhus University, Olof Palmes Allé 43-45, 8200 Aathus, Denmark

**Keywords:** Fetal overgrowth, Placenta, Gene methylation, Insulin, Leptin, Adiponectin

## Abstract

**Background:**

Fetal overgrowth “programs” an elevated risk of type 2 diabetes in adulthood. Epigenetic alterations may be a mechanism in programming the vulnerability. We sought to characterize genome-wide alterations in placental gene methylations in fetal overgrowth and the associations with metabolic health biomarkers including leptin, adiponectin and fetal growth factors.

**Results:**

Comparing genome-wide placental gene DNA methylations in large-for-gestational-age (LGA, an indicator of fetal overgrowth, *n* = 30) versus optimal-for-gestational-age (OGA, control, *n* = 30) infants using the Illumina Infinium Human Methylation-EPIC BeadChip, we identified 543 differential methylation positions (DMPs; 397 hypermethylated, 146 hypomethylated) at false discovery rate < 5% and absolute methylation difference > 0.05 after adjusting for placental cell-type heterogeneity, maternal age, pre-pregnancy BMI and HbA1c levels during pregnancy. Twenty-five DMPs annotated to 20 genes (*QSOX1, FCHSD2, LOC101928162, ADGRB3, GCNT1, TAP1, MYO16, NAV1, ATP8A2, LBXCOR1, EN2, INCA1, CAMTA2, SORCS2, SLC4A4, RPA3, UMAD1,USP53, OR2L13 and NR3C2*) could explain 80% of the birth weight variations. Pathway analyses did not detect any statistically significant pathways after correcting for multiple tests. We validated a newly discovered differentially (hyper-)methylated gene-visual system homeobox 1 (*VSX1*) in an independent pyrosequencing study sample (LGA 47, OGA 47). Our data confirmed a hypermethylated gene—cadherin 13 (*CDH13*) reported in a previous epigenome-wide association study. Adiponectin in cord blood was correlated with its gene methylation in the placenta, while leptin and fetal growth factors (insulin, IGF-1, IGF-2) were not.

**Conclusions:**

Fetal overgrowth may be associated with a large number of altered placental gene methylations. Placental *VSX1* and *CDH13* genes are hypermethylated in fetal overgrowth. Placental *ADIPOQ* gene methylations and fetal circulating adiponectin levels were correlated, suggesting the contribution of placenta-originated adiponectin to cord blood adiponectin.

**Supplementary Information:**

The online version contains supplementary material available at 10.1186/s13148-022-01412-6.

## Background

Fetal overgrowth, as indicated by high birth weight or large-for-gestational-age (LGA), is associated with elevated risks of metabolic syndrome and type 2 diabetes in adulthood [1–3]. The fetus may adapt to adverse environmental cues during gestation that may have long-lasting impact on the vulnerability to a number of chronic diseases [4, 5]—a phenomenon known as developmental “programming.” Such programming in fetal growth restriction may be partly attributable to reduced β-cell mass [6], whereas less is understood about the programming mechanisms in excessive fetal growth.

Epigenetic changes especially in DNA methylation regulating gene expression play a central role in fetal development [7] and may be a mechanism in developmentally programming the vulnerability to metabolic syndrome related disorders. During fetal development, the placenta, through the production of various enzymes and hormones, plays an important role in regulating fetal growth and development [8]. Emerging studies in humans have associated birth weight with placental DNA methylation, but most of these studies have been focused on gene-specific methylation changes in the placentas in small-for-gestational-age (SGA) newborns. We are aware of only two small studies (*n* < 20) [9, 10] on differentially methylated genes in LGA in the placenta. No studies have assessed the associations between LGA-associated placental differential gene methylations and fetal circulating levels of metabolic health biomarkers. Therefore, we sought to evaluate placental gene DNA methylation alterations in LGA, and explore the associations with fetal circulating (cord blood) metabolic health biomarkers including leptin, adiponectin and fetal growth factors [insulin, insulin-like growth factor I (IGF-I) and IGF II].

## Results

### Characteristics of study subjects

Table 1 presents maternal and neonatal characteristics of the 30 pairs of study subjects. Comparing LGA versus birth weight optimal-for-gestational-age (OGA) control subjects, there were no significant differences in maternal age, ethnicity, parity, education, smoking, gestational hypertension, family history of hypertension, family history of diabetes, maternal blood glycated hemoglobin (HbA1c) levels during the 2nd and 3rd trimesters of pregnancy, and gestational age at delivery. Women bearing a LGA fetus had higher pre-pregnancy body mass index (BMI mean: 24.0 vs. 22.0 kg/m^2^) and were more likely to have a cesarean section delivery (73.3% vs. 26.7%). As expected, average birth weight and birth length were substantially higher in LGA vs. OGA newborns.

**Table 1:** Characteristics of study subjects in a matched (1:1) study of 30 pairs of term placentas in LGA and OGA newborns in the Shanghai birth cohort

	LGA (*n* = 30)	OGA(*n* = 30)	P*
*Mothers*			
Age (years)	29.5 ± 3.5	29.8 ± 3.2	0.78
> 35 y	2 (6.7)	2 (6.7)	1.00
Ethnicity, Han	30 (100.0)	30 (100.0)	1.00
Education (university)	19 (63.3)	20 (66.7)	0.79
Primiparity	24 (60.0)	24 (60.0)	1.00
Pre-pregnancy BMI (kg/m2)	24.0 ± 3.4	21.2 ± 3.8	**0.006**
Obesity (BMI > 28)	5 (19.2)	2 (7.4)	0.20
Gestational hypertension	3 (10.0)	0 (0.0)	0.08
Family history of diabetes	4 (13.3)	2 (6.7)	0.39
Family history of hypertension	10 (33.3)	14 (46.7)	0.29
Smoking in pregnancy	1 (3.3)	0 (0.0)	0.31
HbA1c in second trimester	5.0 ± 0.5	5.0 ± 0.3	0.66
HbA1c in third trimester	5.1 ± 0.4	5.1 ± 0.3	0.77
*Newborns*			
Cesarean delivery	22 (73.3)	8 (26.7)	** < 0.001**
Sex, male	17 (56.7)	17 (56.7)	1.00
Gestational age (weeks)	39.5 ± 0.8	39.6 ± 0.8	0.66
Preterm birth (< 37 weeks)	0 (0.0)	0 (0.0)	
Birth weight (g)	4276.2 ± 340.7	3390.2 ± 264.7	** < 0.001**
Z score	2.37 ± 0.85	0.13 ± 0.66	** < 0.001**
Birth length (cm)	51.14 ± 0.77	50.05 ± 0.68	** < 0.001**
Z score	1.12 ± 0.78	0.03 ± 0.71	** < 0.001**
Cord blood biomarkers			
Insulin (pmol/L)	33.6 ± 26.3	40.1 ± 43.4	0.48
Proinsulin (pmol/L)	32.5 ± 32.0	24.4 ± 22.1	0.26
C-Peptide (pmol/L)	291.0 ± 168.9	286.8 ± 161.1	0.92
IGF-I (ng/ml)	88.8 ± 30.4	68.6 ± 22.9	**0.006**
IGF-II (ng/ml)	196.8 ± 28.5	191.4 ± 32.8	0.51
Leptin (ng/ml)	12.4 ± 8.5	9.4 ± 6.7	0.15
Adiponectin, HMW (μg/mL)	14.6 ± 7.0	20.5 ± 10.4	**0.01**
Adiponectin, Total (μg/mL)	37.0 ± 16.8	40.6 ± 18.4	0.43

Data presented are n (%) for categorical variables and mean ± SD for continuous variables

*LGA* large-for-gestational-age (birth weight > 90th percentile); *OGA* optimal-for-gestational-age (birth weight 25th–75th percentiles); *BMI* body mass index, IGF-I insulin-like growth factor I, IGF-II insulin-like growth factor II, HMW, high molecular weight

*P values in t tests for differences in means (for continuous variables) or chi-square tests for differences in proportions (categorical variables) between the two groups. *P* values for biomarkers were from paired *t* tests. *P* values in bold: *P* < 0.05

LGA newborns had significantly higher cord blood IGF-I concentrations (mean: 88.8 vs.68.6 ng/mL, *P* = 0.006), and lower HMW adiponectin concentrations (14.6 vs. 20.5 μg/mL, *P* = 0.014) (Table 1). There were no significant differences in cord blood insulin, C-peptide, proinsulin, leptin, IGF-II and total adiponectin concentrations.

### Differentially methylated positions (DMPs)

**Fig. 1:**
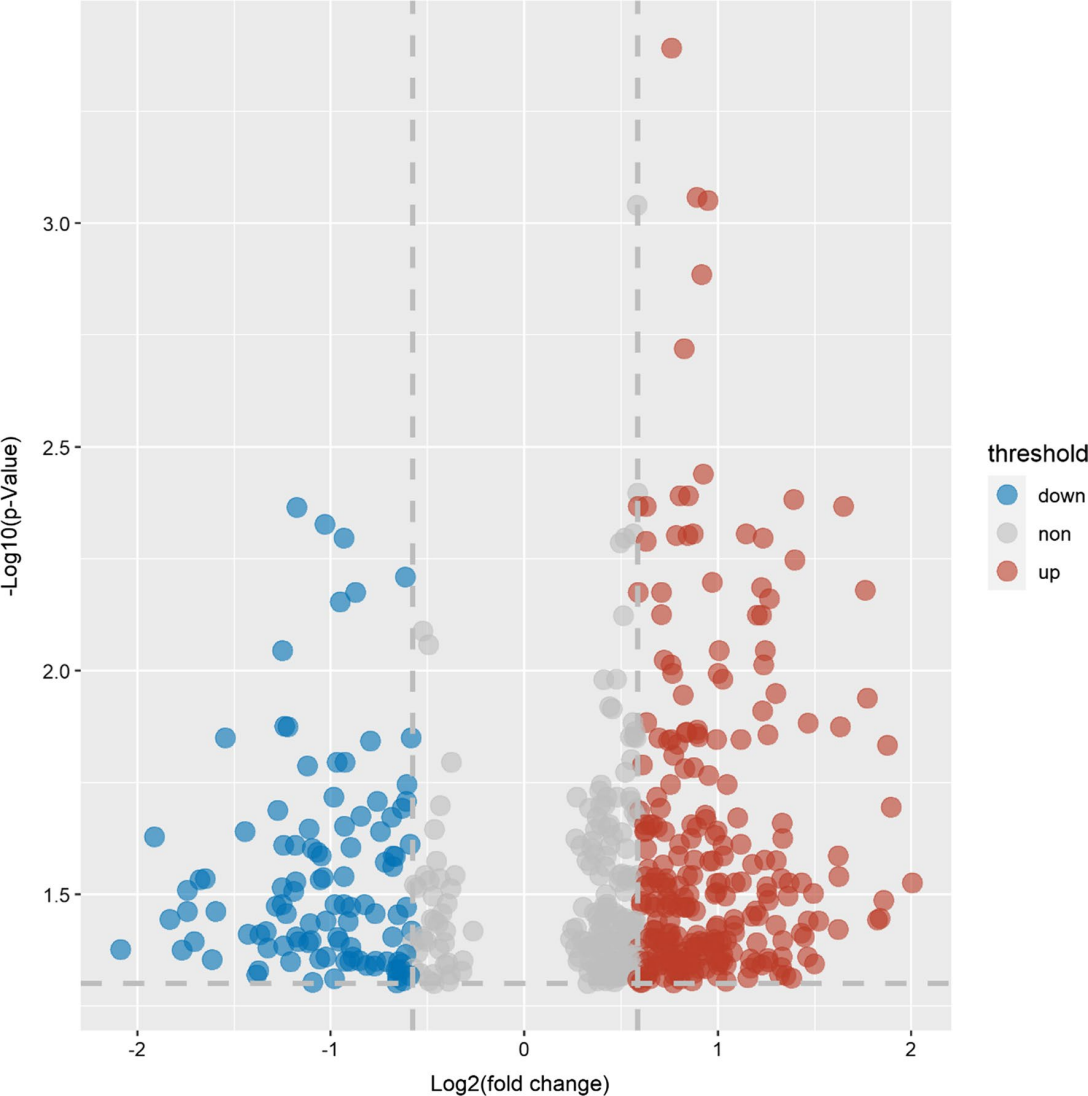
Volcano plot of differentially methylated positions (DMPs) in placental gene DNAs comparing large-for-gestational-age (LGA, birth weight > 90th percentile) versus optimal-for-gestational-age (OGA, 25–75th percentiles, control) newborns. DMPs in the upper left and right quadrants (colored) are differentially methylated at false discovery rate (FDR) < 5%

Adjusting for maternal age, pre-pregnancy BMI, whole blood HbA1c levels at the second and third trimesters of pregnancy and the identified four principal components (from principal component analysis) representing placental cell types (other co-variables were excluded since they were similar and did not affect the comparisons), a total of 543 CpG sites were differentially methylated positions (DMPs) comparing LGA and OGA groups accounting for multiple tests with false discovery rate (FDR) < 5% and absolute methylation difference (delta beta) > 0.05, including 397 hypermethylated and 146 hypomethylated DMPs (Fig. 1, Additional file 2: Table S1). These loci were distributed over 316 genes (232 hypermethylated genes, 84 hypomethylated genes).

The top 50 DMPs (25 hypermethylated, 25 hypomethylated CpG sites) are presented in Tables 2 and 3. The hypermethylated loci were annotated to 14 genes (*EN2, LOC283999, CADM2, ADGB, KRTAP13-4, CRMP1, GFRA1, NRXN1, VSX1, PPFIA2, PLXNC1, DNAJB5, DAOA and ZPLD1*). The hypomethylated sites were annotated to 13 genes (*FAM155A, C21orf34, WNT5B, DTNA, OPRM1, SORCS3, KIF26B, SLCO3A1, KIF26B, LOC284930, SLIT3, NXPH1 and HLA-L*).

**Table 2:** Top 25 hypermethylated sites in placental DNAs in LGA versus OGA newborns

CpG	Gene	Gene Group	Adjusted P	Avg_ LGA	Avg_ Con	Delta Beta
cg22733133	EN2	Body	0.045	0.53	0.34	0.19
cg09473315	LOC283999	Body	0.038	0.32	0.14	0.18
cg20159490			0.040	0.70	0.52	0.18
cg27655158			0.045	0.34	0.17	0.17
cg20065768			0.036	0.66	0.50	0.16
cg11700298	**CADM2**	5′UTR; Body	0.036	0.76	0.60	0.16
cg17470674			0.004	0.82	0.66	0.16
cg01359081	ADGB	Body	0.014	0.47	0.31	0.16
cg17722823	KRTAP13-4	TSS200	0.045	0.51	0.36	0.15
cg09562045	CRMP1	Body;TSS200	0.033	0.28	0.13	0.15
cg27295595			0.044	0.60	0.45	0.15
cg06039355	GFRA1	5′UTR;TSS1500;	0.026	0.44	0.30	0.14
cg06292076			0.038	0.44	0.30	0.14
cg23524195	GFRA1	TSS1500	0.039	0.30	0.17	0.13
cg15134685	NRXN1	1stExon;5'UTR;Body	0.043	0.38	0.25	0.13
cg08045176			0.038	0.61	0.48	0.13
cg17650274	**VSX1**	TSS1500	0.018	0.54	0.41	0.13
cg20926288	PPFIA2	5′UTR	0.032	0.82	0.69	0.13
cg13565656	PLXNC1	1stExon	0.049	0.28	0.15	0.13
cg22916722			0.015	0.23	0.10	0.13
cg14645317	DNAJB5	TSS1500	0.045	0.42	0.29	0.13
cg01735366			0.030	0.79	0.66	0.13
cg14480194			0.006	0.38	0.26	0.12
cg25623522	DAOA	TSS1500;	0.031	0.71	0.58	0.12
cg00657674	ZPLD1	3′UTR	0.027	0.62	0.50	0.12

LGA, large-for-gestational-age (birth weight > 90th percentile); OGA = optimal-for-gestational-age (birth weight 25th–75th percentiles)

The gene name in bold, the CpG site was selected in the pyrosequencing validation study

**Table 3:** Top 25 hypomethylated sites in placental DNAs in LGA vs. OGA newborns

CpG	Gene	Gene Group	Adjusted P	Avg_ LGA	Avg_ OGA	Delta Beta
cg01799062	FAM155A	Body	0.042	0.49	0.65	− 0.16
cg22120852			0.039	0.58	0.73	− 0.15
cg10068793	C21orf34	TSS1500	0.036	0.53	0.68	− 0.15
cg02824489	WNT5B	Body	0.036	0.65	0.79	− 0.14
cg20509944	DTNA	TSS200;5'UTR	0.034	0.54	0.68	− 0.14
cg14348757	OPRM1	1stExon; Body	0.034	0.31	0.45	− 0.14
cg03917817			0.037	0.56	0.69	− 0.13
cg15489003			0.029	0.26	0.39	− 0.13
cg17718664	SORCS3	TSS1500;Body	0.016	0.48	0.61	− 0.13
cg22492024			0.033	0.45	0.57	− 0.12
cg11912591	KIF26B	Body	0.044	0.25	0.37	− 0.12
cg10500641			0.025	0.71	0.83	− 0.13
cg09801894			0.040	0.66	0.78	− 0.12
cg06631347	SLCO3A1	Body	0.045	0.71	0.83	− 0.12
cg08886301	KIF26B	Body	0.025	0.35	0.46	− 0.11
cg25379995	LOC284930	Body	0.029	0.52	0.63	− 0.11
cg02255236	SLIT3	Body	0.040	0.81	0.92	− 0.11
cg10443049	NXPH1	Body	0.040	0.18	0.29	− 0.11
cg27538194			0.049	0.43	0.54	− 0.11
cg10104921			0.045	0.50	0.61	− 0.11
cg17512353	**HLA-L**	Body	0.034	0.52	0.62	− 0.10
cg03370491			0.013	0.54	0.64	− 0.10
cg07409443			0.035	0.44	0.54	− 0.10
cg13306032			0.039	0.21	0.31	− 0.10
cg10358197			0.048	0.46	0.56	− 0.10

LGA, large-for-gestational-age (birth weight > 90^th^ percentile); OGA = optimal-for-gestational-age (birth weight 25^th^-75^th^ percentiles)

The gene name in bold, the CpG site was selected in the pyrosequencing validation study

Our placental epigenome data validated that the cadherin 13(*CDH13*) gene was hypermethylated in LGA as reported in a previous epigenome-wide association study [10] (our study: *CDH13* methylation increased by 0.05 in LGA; the previous study: *CDH13* methylation increased by 0.21 in LGA, according to the publication and communications with the corresponding author).

### Pyrosequencing validation study

We sought to validate a few differentially methylated CpG sites with relatively large methylation differences between LGA and OGA groups in the epigenome-wide association analysis. The study subjects were an independent random sample of 47 pairs of LGA and OGA newborns matched by sex and gestational age from the Shanghai birth cohort (Additional file 3: Table S2). Women bearing a LGA fetus had higher pre-pregnancy BMI and were more likely to be have a cesarean section delivery, while other maternal characteristics were similar in LGA and OGA groups. As expected, average birth weight and birth length were substantially higher in LGA versus OGA newborns.

Three CpG sites were selected among the top 25 DMPs in the pyrosequencing validation study. The CpG site (cg11700298) annotated to cell adhesion molecule 2 (*CADM2*)] was selected because polymorphism in this gene has been associated with obesity and type 2 diabetes [11, 12]. We randomly selected 2 more CpG sites among the top 25 DMPs in the validation study. They were cg17650274 [annotated to gene visual system homeobox 1 (*VSX1*)] and cg17512353 [annotated to gene major histocompatibility complex, class I, L (*HLA-L*)]. The pyrosequencing validation vs. epigenome-wide study results for the 3 DMPs are shown in Additional file 3: Table S3; only one DMP (*VSX1* gene) was validated in the pyrosequencing study.

### GO and KEGG pathways

The GO analysis showed that DMPs were mostly enriched in axon development (biological processes), motile cilium (cellular components), metal-ion transmembrane transporter activity (molecular functions) (Additional file 2: Table S4). KEGG pathway analysis showed that DMPs might mainly be involved in 9 pathways including GnRH secretion, protein digestion and absorption (Additional file 2: Table S5). However, after correction for multiple tests, the results were not statistically significant for all pathways.

### Ingenuity pathway analysis (IPA)

IPA identified 16 canonical pathways **(**Fisher^’^s exact crude *P* < 0.05, Additional file 2: Table S6). None of the pathways achieved statistical significance after Benjamini–Hochberg correction for multiple tests. Differentially methylated genes were most enriched in the G-Protein Coupled Receptor Signaling (9 genes, crude *P* < 0.05). There was also some evidence of enrichment (crude *P* < 0.05) in the Pentose Phosphate (Oxidative Branch) and Estrogen Biosynthesis pathways.

### Differentially methylated regions (DMRs)

To reduce data dimensionality and identify differential methylations over gene areas, we analyzed the methylation data for differentially methylated regions (DMRs) by DMRcate and comb-p. The DMRcate program identified 135 DMRs at FDR < 5% between LGA and OGA groups (Additional file 2: Table S7), and these DMRs were annotated to 94 genes, while the other DMRs were located in the OpenSea areas. The comb-p program identified 31 DMRs with Sidak corrected *P* < 0.05 (Additional file 2: Table S8), 15 DMRs were annotated to genes, while the other DMRs were located in the OpenSea areas. Three DMRs were identified by both DMRcate and comb-p, and these loci were annotated to 3 genes (*PRMT2, BACE1 and TRAK2*; Table 4).

**Table 4:** Placental DNA differentially methylated regions (DMRs) in LGA vs. OGA newborns identified in both comb-p and DMRcate package analyses

DMR	CpGs of the DMR	Direction of association	Nearby gene	Gene group
Chr21:48,078,809–48,079,700	cg01123035, cg22256816	+ / +	PRMT2	Body
chr11:117,166,784–117,167,825	cg22974118, cg03026698, cg27313579, cg01814586, cg07189750	± / + / ±	BACE1	TSS1500;Body
Chr2:202,286,912–202,287,373	cg01561344, cg16375762	− / +	TRAK2	5'UTR

LGA, large-for-gestational-age (birth weight > 90th percentile); OGA = optimal-for-gestational-age (birth weight 25^th^–75th percentiles)

### Correlations of DMPs with birth weight and cord blood biomarkers

Among the 543 DMPs, 494 CpG sites were correlated with birth weight z score (crude *P* < 0.05), and the correlations for 486 DMPs remained statistically significant after correction for multiple tests (Additional file 2: Table S9). These 486 DMPs were annotated to 286 genes. In LASSO regression identifying the most important DMPs, 25 DMPs were selected and could explain 80.2% of the variations in birth weight (z) (Additional file 2: Table S10). These 25 DMPs were annotated to 20 genes (*QSOX1, FCHSD2, LOC101928162, ADGRB3, GCNT1, TAP1, MYO16, NAV1, ATP8A2, LBXCOR1, EN2, INCA1, CAMTA2, SORCS2, SLC4A4, RPA3, UMAD1,USP53, OR2L13 and NR3C2*) (Additional file 2: Table S10).

Additional file 2: Table S11 presents the correlations of DMPs with cord blood insulin, proinsulin, C-peptide, IGF-II, IGF-I, leptin, total or HMW adiponectin**.** All these correlations did not reach statistical significance after Benjamini and Hochberg correction for multiple tests.

### Gene-specific correlations of placental DNA methylations and cord blood biomarkers

Additional file 3: Table S12 presents the gene-specific correlations of placental gene CpG sites and cord blood biomarkers. For INS-IGF2/IGF2AS/IGF2, methylation levels in four CpG sites (cg08014499, cg21728792, cg05203776 and cg22225943) were positively correlated with cord blood insulin (*r* = 0.26 to 0.28, crude *P* = 0.03 to 0.05), and in one CpG site positively correlated with cord blood C-peptide (cg17434309, *r* = 0.28, crude *P* = 0.03), proinsulin (cg17434309, *r* = 0.31, crude *P* = 0.02) and IGF-II (cg23889607, *r* = 0.30, crude *P* = 0.02). Methylation levels in four CpG sites (cg13928782, cg21574853, cg07096953 and cg20088847) were negatively correlated with cord blood IGF-II (*r* = − 0.30 to − 0.35, crude *P* = 0.007 to 0.024), and in one CpG site negatively correlated with cord blood proinsulin (cg13670288, *r* = − 0.26, crude *P* = 0.049). All these correlations, however, did not reach statistical significance after correction for multiple tests.

Several correlations between *ADIPOQ* gene methylation levels and cord blood total or HMW adiponectin remained statistically significant after correction for multiple tests. Total adiponectin was correlated with cg16126291 (*r* = − 0.35, adjusted *P* = 0.03), cg02235049 (*r* = − 0.32, adjusted *P* = 0.047), cg10681525 (*r* = − 0.40, adjusted *P* = 0.01) and cg18537894 (*r* = 0.40, adjusted *P* = 0.01). HMW adiponectin was correlated with cg16126291 (*r* = − 0.42, adjusted *P* = 0.01) and cg18537894 (*r* = 0.36, adjusted *P* = 0.036).

## Discussion

### Main findings

We observed 543 DMPs in placental DNA in LGA—an indicator of fetal overgrowth, and identified 25 DMPs annotated to 20 genes that could explain the majority of birth weight variations. We validated that the *VSX1* gene was hypermethylated in fetal overgrowth in an independent pyrosequencing study sample, and confirmed a hypermethylated gene (*CDH13*) in fetal overgrowth reported in a previous epigenome-wide association study. Three DMRs were identified and annotated to three genes (*PRMT2, BACE1 and TRAK2*). We did not detect any specific significant pathway after correction for multiple tests. Placental gene methylation and fetal circulating hormone biomarkers were correlated for adiponectin, but not for leptin and fetal growth factors.

### Data interpretation and comparisons with findings in previous studies

Our study is the largest in assessing genome-wide placental DNA methylations in LGA. A previous study in 27 LGA and 19 appropriate for gestational age (AGA) controls observed no gene methylation alterations in LGA in an epigenome-wide association analysis of cord tissue DNA methylations using the Infinium Human Methylation 450 K BeadChips [13]. Another epigenome-wide association study (5 LGA, 6 AGA controls) reported no differences in placental DNA methylation levels using reduced representation bisulfite sequencing [10]. However, a large study (*n* = 1023) assessing placental global DNA methylation by LC–MS/MS demonstrated that LGA displayed significantly higher global placental DNA methylation compared to AGA [14]. Another study (6 LGA and 6 AGA controls) reported that among 17,244 methylation variable positions, 705 were hypermethylated (> 1.7-fold) and 351 were hypomethylated (< 0.5-fold) in LGA in placental DNA methylations using the Infinium Human Methylation 850 K BeadChips [9]. These results are consistent with our data: Hypermethylated sites (*n* = 397) are more frequent than hypomethylated sites (*n* = 146) in LGA.

### DMPs

We identified 486 DMPs in placental gene DNA comparing LGA versus controls. Many of these DMPs (165/486) demonstrated weak-to-moderate correlations (*r*: 0.25 to 0.45) with a fetal growth factor (IGF-I, IGF-II, insulin), leptin or adiponectin in cord blood in crude correlation analyses (Additional file 2: Table S11), but none reached statistical significance after correction for multiple tests. The placenta is a transient fetal organ, and its gene expression might affect fetal growth through influencing the function of the placenta, rather than through affecting fetal circulating levels of growth factors. This may explain the lack of correlation between placental IGF-I gene methylations and circulating/fetal IGF-I concentrations, because IGF-I is produced in fetal liver cells only. Gene methylation patterns are tissue-specific. A specific gene’s methylation pattern in the placenta might unlikely reflect the gene’s methylation pattern in other fetal tissues especially if the gene’s expression is tissue-specific. For genes that are expressed in multiple tissues, one might speculate that some methylation patterns may be shared across multiple tissues. It is unknown which gene’s specific methylation patterns in the placenta are present in other fetal tissues. The implications of placental gene methylation signatures for short- and long-term metabolic health in the offspring are largely unknown and remain to be understood. Emerging evidence suggests the prognostic value of placental gene methylations on long-term metabolic health in children: Placental lipoprotein lipase gene DNA methylation alterations have been correlated with fat mass in children at age 5 years [15].

Notably, 25 DMPs annotated to 20 genes (*QSOX1, FCHSD2, LOC101928162, ADGRB3, GCNT1, TAP1, MYO16, NAV1, ATP8A2, LBXCOR1, EN2, INCA1, CAMTA2, SORCS2, SLC4A4, RPA3, UMAD1, USP53, OR2L13 and NR3C2*) could explain over 80% of the birth weight variations. Some of these genes have been implicated in the regulation of glucose homeostasis, β cell function, adipose tissue or muscle growth. *FCHSD2* and *SLC4A4* have been associated with β cell function[16, 17], while *USP53* has been related to adiposity homeostasis [18]. *SLC4A4* knockout mice were protected from diet-induced metabolic stress and β cell dysfunction [17] Loss of *FCHSD2* was associated with impaired insulin secretion in a human-derived β cell study [16]. Elevated *USP53* gene RNA expression in adipose tissue has been associated with good weight control in obese subjects [18]. The *QSOX1* gene has been implicated in cortical bone accrual and strength in mice [19]. The *ADGRB3* gene may be involved in myoblast fusion in the muscle of vertebrates [20] and implicated in insulin secretion from pancreatic β-cells [21]. Adipocyte-specific overexpression of *NR3C2* exacerbates metabolic syndrome in mice [22]. However, there have been no reports on whether these genes are correlated with fetal growth in humans or animals. Our data on placental gene methylations suggest the importance of these 20 genes for fetal growth, probably through their impacts on placental function that deserves further mechanistic studies. A lack of replicable findings in genome-wide association studies is a common problem in data interpretation concerning the robustness of positive findings. It is noteworthy that our data validated a hypermethylated gene (*CDH13*) reported in a previous epigenome-wide association study [10].

*CDH13* (cadherin 13), a 95 kd glycoprotein, is an atypical member of the cadherin family of cell adhesion molecules [23]. *CDH13* may serve as an adiponectin receptor and has been associated with plasma adiponectin and the risk of type 2 diabetes [24, 25]. *CDH13* levels in adipose tissue and the circulation are decreased in obese mice and humans and are restored by weight loss in humans [26]. Therefore, *CDH13* is considered as a marker of fat tissue plasticity that might reflect the health status of adipose tissue. We observed that *CDH13* was hypermethylation in LGA, consistent with the results in a previous study [10]. Tyrberg and colleagues reported T-cadherin (*CDH13*) as a novel component of insulin granules, suggesting that it might contribute to the regulation of insulin secretion independently of adiponectin [27]. It would be interesting to determine whether altered placental *CDH13* gene hypermethylation may be an epigenetic biomarker of the elevated risk of metabolic dysfunctional disorders in LGA subjects in long-term follow-up studies.

Placental *VSX1* gene was observed to be hypermethylated in LGA in both the epigenome-wide association analysis and pyrosequencing validation study. We failed to confirm the other two DMPs identified from the epigenome-wide association analysis in the pyrosequencing validation study, underscoring the uncertain nature of genome-wide discovery research. Despite best efforts in accounting for potential confounding effects and biases, unmeasured confounding effects might weaken the power of genome-wide association studies in identifying the true differences.

The *VSX1* (visual system homeobox 1) gene encodes a paired-like homeodomain transcription factor and is associated with eye development [28]. *VSX1* gene variants may play an important role in the development of keratoconus [29]. Our data suggest hypermethylation of placental *VSX1* gene in fetal overgrowth, and long-term follow-up studies are required to determine its potential significance as an epigenetic biomarker for metabolic health in later life.

### DMRs

Three DMRs in LGA were identified in both DMRcate and comb-p analyses, which were annotated to three metabolic health relevant genes—*BACE1, TRAK2* and *PRMT2*.

*BACE1* (β-site APP-cleaving enzyme 1) is expressed in pancreas, liver and skeletal muscle [30]. *BACE1* may play an important role in glucose metabolism; *BACE1*-deficient liver and skeletal muscle exhibit improved insulin sensitivity and glucose homeostasis in mice [31]. Neuronal human *BACE1* knock-in in mice induced systemic diabetes [32]. High glucose levels might upregulate *BACE1* expression via ROS generation in SK-N-MC cells [33]. Moreover, the *BACE1* gene polymorphism has been associated with the risk of diabetes in PIMA Indians [34]. Thus, alterations in *BACE1* levels may be involved in the pathophysiology of diabetes.

Trafficking protein kinesin binding 2 (*TRAK2*) is a regulator of protein and organelle trafficking through its role as a kinesin and dynein binding protein, and it may function in neuronal mitochondrial trafficking [35]. *TRAK2* has been reported as a novel regulator of ATP-binding cassette, sub-family A member 1 (*ABCA1*) expression, cholesterol efflux and HDL biogenesis, and therefore, *TRAK2* may be an important target in the treatment of cardiovascular disorders [36].

Protein arginine methyltransferase 2 (*PRMT2*)—a type I enzyme, contains a highly conserved catalytic Ado-Met binding domain and unique Src homology (SH) 3 domain that binds proteins with proline-rich motifs [37]. Genetic deletion of *PRMT2* has been associated with a lean, leptin-hypersensitive “anti-diabetes-like” phenotype in mice [38].

Our results suggest altered methylations in placental *PRMT2, BACE1* and *TRAK2* genes in fetal overgrowth. It remains to be determined whether they could be promising epigenetic biomarkers of the increased risk of metabolic dysfunctional disorders in LGA subjects in later life.

### Pathways

The IPA results suggest the enrichment of three genes in the “Pentose Phosphate” and “Estrogen Biosynthesis” pathways. A smaller study (6 LGA and 6 controls) in cord blood DNA reported 27 genes enriched in “Diseases and Disorders” terms [39] without pathway analysis. There were no consistent findings between our GO and KEGG pathway results and those reported in a previous study [9]. To be noted, all pathways did not reach statistical significance after correction for multiple tests in our study. Previous studies did not adjust for multiple tests. Larger studies are warranted to clarify whether any specific pathway may be affected in placental gene methylations in LGA.

### Associations with cord blood biomarkers

Placental gene methylations were correlated with fetal circulating (cord blood) hormone levels for adiponectin, but not for leptin and fetal growth factors after correction for multiple tests. Bouchard and colleagues reported that placental *ADIPOQ* gene methylation levels were negatively correlated with maternal circulating adiponectin concentrations [40]. We are unaware of any report on placental *ADIPOQ* methylation and cord blood adiponectin. In our data, total adiponectin was inversely correlated with *ADIPOQ* gene methylations at 3 CpG sites (cg16126291, cg02235049 and cg10681525), and HMW adiponectin was negatively correlated with methylations at 2 CpG sites (cg16126291 and cg02235049). Fetal/cord blood adiponectin levels are attributable to adiponectin secretion by both fetal adipose tissue (brown adipocytes) and vascular cells [41–43]. Adiponectin is highly expressed in vascular endothelial cells of fetal capillaries [41]. There is no evidence of maternal origin of adiponectin in fetal circulation [42]. It remains controversial whether the human placenta secrets adiponectin [44]. The placenta is a fetal tissue rich in blood vessels and capillaries which may explain the placental “production” of adiponectin. In our study, cord blood adiponectin concentrations were negatively correlated with multiple placental *ADIPOQ* gene methylation sites, suggesting the contribution of placenta-originated adiponectin in cord blood adiponectin.

## Limitations

There were several study limitations. First, the sample size was relatively modest (but still the largest placenta epigenome study on LGA thus far). The study might be under-powered to detect small differences, and we could not rule out the possibility of false positive findings which may be present in all (epi)genome-wide association studies. This might explain the lack of consistent findings in previous (epi)genome-wide association studies and that only one of the three selected DMPs was validated in an independent sample. Large studies are warranted to validate the findings. Second, the DNA isolated from placental tissues comes from multiple cell types that might have not been adequately accounted for using the principal component analysis. This may limit the capacity to identify the true differences. Third, the study was limited to Chinese subjects. More studies in other ethnic groups are warranted to understand the generalizability of the study findings.

## Conclusions

Fetal overgrowth appears to be associated with altered methylations in a large number of placental genes. Placental *CDH13* and *VSX1* genes are hypermethylated in fetal overgrowth. Long-term follow-up studies are required to determine whether these differentially methylated genes may be promising epigenetic biomarkers of the elevated risk of metabolic dysfunctional disorders in later life in subjects with fetal overgrowth. Placental *ADIPOQ* gene methylations and fetal circulating adiponectin levels were correlated, suggesting the contribution of placenta-originated adiponectin in cord blood adiponectin.

## Methods

### Study design

We conducted a nested case–control study in the Shanghai birth cohort (SBC) [45]. LGA was defined as birth weight > 90th percentile, according to the Chinese sex- and gestational age-specific birth weight standards [46]. Controls were optimal-for-gestational-age (OGA, birth weight 25th-75th percentiles) newborns. Each LGA was matched (1:1) to an OGA newborn by sex and gestational age (within 7 days) at delivery. The study subjects (30 pairs of LGA/OGA) were randomly sampled from all eligible LGA and OGA newborns in the SBC. All cases and controls were term births with normal Apgar score (> 7), and all mothers were free of severe chronic diseases before pregnancy (e.g., essential hypertension, type 1 or 2 diabetes), severe pregnancy complications (e.g., preeclampsia) or life-threatening conditions.

### Placenta and cord blood samples

Trained research staff collected cord blood and placental tissue samples following standardized operating procedures. Each of the four placenta quadrants was sampled approximately 1.5 cm away from the umbilical cord insertion from the fetal side of the placenta. Fetal membranes and visible large vessels were removed, and phosphate-buffered saline was used to wash placenta samples before separating into maternal- and fetal-side samples. Placental and cord blood samples were kept at 4℃ in a refrigerator between 0 and 4 h before stored at − 80 °C in a freezer until DNA extraction. There were no reports of specimen handling protocol violations. Genomic DNA was extracted from fetal-side placental samples using DNeasy & Tissue Kit (Qiagen) following the manufacturer’s manual. Purity was examined by measuring the A_260:_A_280_ ratio (mean ± SD: 1.89 ± 0.02; range 1.85–1.98).

All collected maternal and cord blood samples (in EDTA tubes for plasma, in tubes without any coagulant for serum) were kept on ice, stored temporarily in a 4 °C refrigerator and centrifuged (4000 r/min for 10 min) within 2 h after the specimen collection. The separated serum and plasma samples were stored in multiple aliquots at − 80 °C until assays.

### Genome-wide DNA methylation measurements

Prior to DNA isolation, placental tissue samples were homogenized for 1 min at 6000 rpm × 3 (5 min on ice in between intervals) in lysis buffer (180 μl buffer ATL with 20 μl proteinase K). Placental DNA was then isolated using the DNAeasy kit (Qiagen, UK, Catalog # 69,504) according to manufacturer’s instructions. DNA (500 ng) was treated with bisulfate using an EZ DNA Methylation Gold kit (Zymo Research, Irvine, CA, Catalog # D5006) according to the manufacturer’s instructions. DNA methylations were measured by Illumina Human Methylation EPIC BeadChip (Illumina, Inc., San Diego, CA, USA), which provides genome-wide coverage containing > 850,000 CpG methylation sites. The experiments followed the manufacturer’s protocol (https://emea.support.illumina.com/content/dam/illumina-support/documents/documentation/chemistry_documentation/infinium_assays/infinium_hd_methylation/infinium-hd-methylation-guide-15019519-01.pdf). Samples were randomly placed in different slides in the experiments.

Package “minfi” was used to import and preprocess raw methylation data. For all the study samples, the proportion of CpG sites with detection *P* value > 0.01 was less than 5%. Thus, all samples were included in subsequent data analyses. We excluded 2,677 CpG sites with detection *P* value > 0.01 in more than 5% of all the samples. Functional normalization was applied to remove between-array (unwanted) variations using control probes [47]. We further excluded 41,710 CpG sites with bead count < 3 in more than 5% of all the samples, 18,487 annotated to sex chromosomes, 83,460 SNPs inside the probe body, in the CpG interrogation site, or at the single nucleotide extension with a minor allele frequency of ≥ 0.05, 33,202 suspected cross-reactive sites [48], and 2301 non-CpG sites, leaving 684,022 CpG sites in subsequent data processing. Beta-mixture quantile dilation (BMIQ) was then applied to adjust for type 2 probe bias [49]. We filtered out sites with average methylation (β value) < 5% or > 95% (*n* = 183,267), as extreme β values tended to have low reproducibility [50], and small-to-moderate changes in methylation levels may not have much biologically significant implications at both extremes. A total of 500,755 CpG sites were retained in the final epigenome-wide association analysis. Potential bias due to different slides was adjusted for using the ComBat function in sva package [51]. The density plot did not reveal significant differences in beta value distributions between LGA and OGA (Additional file 1: Figure S1). Beta values were transformed to M values using lumi package in differential methylation analysis since M values had better statistical performance [52]. Beta values were presented for quantifying the differences in methylation levels between groups for data interpretation.

### Pyrosequencing validation study

We sought to validate several DMPs identified in the genome-wide discovery analysis. The study subjects were an independent random sample of 47 pairs of LGA-OGA (control) subjects from the Shanghai birth cohort. Placental DNA was sodium-bisulfite treated using the EZ-96 DNA Methylation-Lighting Kit (Zymo Research, Irvine, CA, Catalog # D5006), and PCR-amplified with primers designed by PyroMark Assay Design software (version 2.0, Qiagen). All procedures were performed according to the manufacturer’s protocols. Pyrosequencing was performed using the PyroMark Q48 system (Qiagen), and cytosine methylation was quantified using the PyroMark Q241.010 software.

### Biochemical assays

Cord serum insulin and insulin-like growth factor 1 (IGF-I) were measured by chemiluminescent assays (ADVIA Centaur and Immulite2000, SIEMENS, Germany). Cord plasma IGF-II was measured by an ELISA kit from R&D system (Minnesota, USA, catalog # DG200), and plasma C-peptide and proinsulin by ELISA kits from Mercodia system (Uppsala, Sweden. catalog # 10–1136-01 for C-peptide, catalog # 10–1118-01 for proinsulin), respectively. Plasma total and high-molecular-weight (HMW) adiponectin were measured by an ELISA kit from ALPCO (Salem, NH, USA, catalog # 47-ADPHU-E01), and plasma leptin by an ELISA kit from Invitrogen (Carlsbad, CA, USA, catalog # KAC2281), respectively. Maternal whole blood HbA1c was measured by high-performance liquid chromatography (BIO-RAD VARIANT II, California, USA). The detection limits were 3.5 pmol/l for insulin, 25 ng/ml for IGF-I, 1.88 pg/ml for IGF-II, 1.7 pmol/l for proinsulin, 25 pmol/L for C-peptide, 0.034 ng/mL for HMW and total adiponectin and 3.5 pg/mL for leptin, respectively. Intra-assay and inter-assay coefficients of variation were in the ranges of 2.0–6.5% for insulin and IGF-I, 5.0–8.6% for proinsulin, 0.4–13.5% for C-peptide and 2.4–9.3% for IGF-II, 6.9%–10.4% for leptin, total and HMW adiponectin, respectively. In all biomarker assays, the laboratory technicians were blinded to the clinical status (LGA or not) of study subjects.

### Statistical analysis

All analyses were conducted in R using the R studio (https://www.rstudio.com/). The association between LGA status and DNA methylation (M-value) in each CpG site was assessed by lmFit function in limma package. For placental cell types, we used the ReFACTor package to select the number of principal components and then included the selected components (k = 4) as the covariates in adjusting for cell-type heterogeneity in differential methylation position (DMP) analyses [53]. The selection of principal components was based on a score calculated as the -log of the ratio of two adjacent eigenvalues [the i-th eigenvalue to the (i-1)-th eigenvalue] in the principal components analysis. The number of principal components was chosen (k = 4) when the score was near 0. The comparisons were adjusted for important covariates including maternal age, pre-pregnancy BMI, and glycosylated hemoglobin levels (HbA1c) during the second and third trimesters of pregnancy. To minimize false discovery findings, p values were adjusted according to Benjamini and Hochberg’s method in correction for multiple tests. DMPs comparing LGA and control groups were selected at false discovery rate (FDR) < 5% and absolute methylation difference (delta beta) > 0.05 (to identify “true” DMPs with differences that are unlikely to be measurement errors). To identify the most important differentially methylated genes that are correlated with birth weight, the Least Absolute Shrinkage and Selection Operator (LASSO)-regression [54] was performed using the “glmnet” package.

To understand the functional roles of the DMPs, we performed Ingenuity Pathway Analysis (IPA) to annotate the significant canonical pathways and performed Gene Ontology (GO) and Kyoto Encyclopedia of Genes and Genomes (KEGG) pathway enrichment analyses using the missMethyl package [55].

At the region-level, differentially methylated regions (DMRs) were identified using the DMRcate package [56] and comb-p [57]. DMRcate package identifies the differentially methylated regions based on tunable kernel smoothing of the signal of methylation changes [56]. As recommended by the authors, a bandwidth of 1000 nucleosides and a scaling factor of 2 were used. Comb-p deals with autocorrelations in neighboring p values and reports region-based p values using Sidak correction for multiple tests. *P* value < 10^–3^ was set to start a region, and a distance of 200 bp was selected to extend the region in the presence of another *P* value < 10^–3^. Significant DMRs were selected at FDR < 5%.

Pearson partial correlations were used to examine the associations of DNA methylations with cord blood biomarkers (leptin, adiponectin, insulin, proinsulin, C-peptide, IGF-I and IGF-II) adjusting for gestational age at birth. First, we assessed the correlations with DNA methylation levels in the corresponding specific genes (*LEP, ADIPOQ, IGF1, INS-IGF2*). Of the 500,755 CpG sites, a total of 21 sites were annotated to *LEP* (chr7: 127,876,829–127,894,849), 12 sites to *ADIPOQ* (chr3: 186,559,147–186,575,325), 12 sites to *IGF1* (chr1:102,795,843–102,875,301) and 119 sites to *INS/INS-IGF2/IGF2* (chr11: 2,150,687–2,183,864). The total numbers of sites remained in the partial correlation analyses were 21 for *LEP*, 12 for *ADIPOQ*, 12 for *IGF1* and 119 for *INS/INS-IGF2/IGF2* genes, respectively. Benjamini–Hochberg’s method was used in calculating the p values in correction for multiple tests. Second, we assessed the correlations of LGA-associated DMPs with fetal growth (birth weight z score) and cord blood biomarkers. CpG sites with crude P values < 0.05 were presented along with the Benjamini–Hochberg corrected P values accounting for multiple tests.

## Supplementary Information

**Additional file 1. Figure S1. **Density plots of beta values in LGA and OGA (control) groups.

**Additional file 2.** Appendix Tables S1, S4, S5, S6, S7, S8, S9, S10 and S11.

**Additional file 3.** Appendix Tables S2, S3 and S12.

## Data Availability

Access to the deidentified participant research data must be approved by the research ethics board on a case-by-case basis; please contact the corresponding authors (zcluo@lunenfeld.ca; jl@clin.au.dk) for assistance in data access request. The EWAS data on placental gene methylations are available at https://www.ncbi.nlm.nih.gov/geo/query/acc.cgi?acc=GSE204977.

## Abbreviations

LGA: Large-for-gestational-age
OGA: Optimal-for-gestational-age
SBC: Shanghai birth cohort
DMP: Differential methylation position
DMR: Differential methylation region
DNA: Deoxyribonucleic acid
BMI: Body mass index
HbA1c: Glycosylated hemoglobin A1c
IGF-I: Insulin-like growth factor I
IGF-II: Insulin-like growth factor II
IPA: Ingenuity pathway analysis
FDR: False discovery rate
