# Supplementary material for: Genome-wide placental DNA methylations in fetal overgrowth and associations with leptin, adiponectin and fetal growth factors
Source: Clin Epigenetics. 2022 Dec 30;14:192. doi: 10.1186/s13148-022-01412-6 (PMC9801645; doi:10.1186/s13148-022-01412-6)
Supplement: Supplementary file 1 — Additional file 1. Figure S1. Density plots of beta values in LGA and OGA (control) groups. [file 13148_2022_1412_MOESM1_ESM.docx]

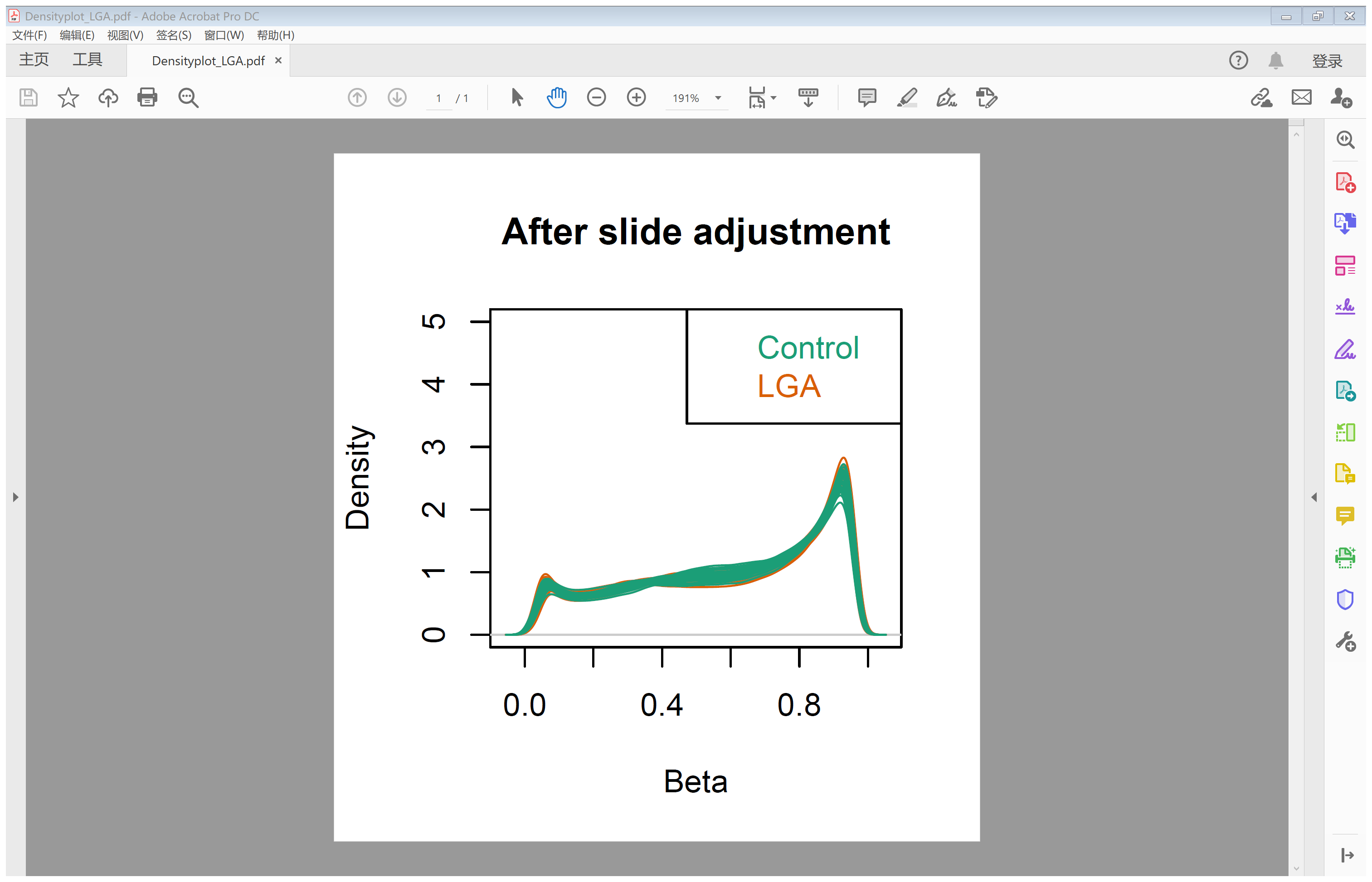


**Figure S1**. Density plots of beta values in LGA and OGA (control) groups in an epigenome-wide association study of placental DNA methylations. LGA, large-for-gestational age (birth weight>90th percentile); OGA, optimal-for-gestational-age (birth weight 25th-75th percentiles).
