# Supplementary material for: Genome-wide placental DNA methylations in fetal overgrowth and associations with leptin, adiponectin and fetal growth factors
Source: Clin Epigenetics. 2022 Dec 30;14:192. doi: 10.1186/s13148-022-01412-6 (PMC9801645; doi:10.1186/s13148-022-01412-6)
Supplement: Supplementary file 3 — Additional file 3. Appendix Tables S2, S3 and S12. [file 13148_2022_1412_MOESM3_ESM.docx]

**Table S2.** Participants’ characteristics in the pyrosequencing validation study in Shanghai Birth Cohort

|  | LGA (n=47) | OGA (n=47) | P * |
| --- | --- | --- | --- |
| Mothers |  |  |  |
| Age (years) | 30.5±4.0 | 29.8±3.4 | 0.32 |
| >35 y | 6 (12.8) | 3 (6.4) | 0.29 |
| Ethnicity, Han | 47 (100.0) | 47 (100.0) | 1.00 |
| Education (university) | 33 (70.2) | 30 (63.8) | 0.51 |
| Primiparity | 37 (78.7) | 38 (80.9) | 0.80 |
| Pre-pregnancy BMI (kg/m2) | 22.9±3.5 | 21.2±2.2 | **0.009** |
| Gestational hypertension | 1 (2.1) | 0 (0.0) | 0.31 |
| Family history of diabetes | 5 (11.1) | 6 (12.8) | 0.81 |
| Family history of hypertension | 14 (31.1) | 20 (42.6) | 0.26 |
| Smoking in pregnancy | 1 (2.1) | 0 (0.0) | 0.31 |
| HbA1c in second trimester | 4.9±0.5 | 4.8±0.3 | 0.25 |
| HbA1c in third trimester | 5.1±0.3 | 5.0±0.3 | 0.30 |
| Newborns |  |  |  |
| Cesarean delivery | 27 (57.5) | 11 (23.4) | **<0.001** |
| Sex, male | 26 (55.3) | 26 (55.3) | 1.00 |
| Gestational age (weeks) | 39.9±1.2 | 39.6±1.0 | 0.26 |
| Preterm birth (<37 weeks) | 2 (4.3) | 1 (2.1) | 0.56 |
| Birth weight (g) | 4183.2±353.7 | 3304.6±280.5 | **<0.001** |
| Z score | 2.13±0.70 | -0.06±0.58 | **<0.001** |
| Birth length (cm) | 51.32±1.09 | 49.64±1.33 | **<0.001** |
| Z score | 1.25±0.99 | -0.31±1.04 | **<0.001** |

Data presented are n (%) for categorical variables and mean±SD for continuous variables.

LGA, large-for-gestational-age (birth weight >90^th^ percentile); OGA= optimal-for-gestational-age (birth weight 25^th^-75^th^ percentiles); BMI, body mass index, IGF-I insulin-like growth factor-I, IGF-II insulin like growth factor-II, HMW, high molecular weight.

*P values in t-tests for differences in means (for continuous variables) or Chi square tests for differences in proportions (categorical variables) between the two groups. P values in **bold**: P<0.05.

**Table S3**. Placental gene DNA methylation levels (%) in three CpG sites in an independent pyrosequencing validation study sample (n=47 pairs of LGA and OGA control newborns) vs. the results in the epigenome-wide association study (n=30 pairs of LGA and OGA control newborns) using the Infinium MethylationEPIC Beadchip

|  |  | **Pyrosequencing validation study** | | |  | **MethylationEPIC Beadchip Study** | | |
| --- | --- | --- | --- | --- | --- | --- | --- | --- |
| **CpG** |  | LGA  (n=47) | OGA  (n=47) | P* |  | LGA  (n=30) | OGA  (n=30) | P^#^ |
|  |  | Mean±SD | Mean±SD |  |  | Mean±SD | Mean±SD |  |
| cg17650274 | VSX1 | 40.75±11.49 | 34.25±15.33 | 0.035 |  | 54.02±10.13 | 41.03±10.81 | <0.0001 |
| cg17512353 | HLA-L | 26.82±15.65 | 23.25±14.82 | 0.24 |  | 51.67±16.33 | 62.09±12.10 | 0.0068 |
| cg11700298 | CADM2 | 43.15±13.86 | 46.50±14.38 | 0.22 |  | 76.37±10.20 | 60.55±19.0 | 0.002 |

*P values from paired t-tests.

^#^P values from generalized linear models adjusting for maternal age, pre-pregnancy-BMI and whole blood glycosylated hemoglobin (HbA1c) levels during the 2^nd^ and 3^rd^ trimesters of pregnancy and placental cell type heterogeneity (4 identified princiapl components); other co-variables were not included since they were similar and did not affect the comparisons.

LGA, large-for-gestational-age (birth weigth >90^th^ percentile, according to the Chinese sex- and gestational age-specific birth weight standards); OGA= optimal-for-gestational-age (birth weight 25^th^-75^th^ percentiles);

**Table S12.** Gene specific correlations between placental DNA methylations and cord blood biomerkers

| Biomarker | CpGsite | r | *P | ^#^Adjusted  P | UCSC_Ref  Gene_Name | UCSC_Ref  Gene_Group |
| --- | --- | --- | --- | --- | --- | --- |
| Insulin | cg08014499 | 0.28 | 0.033 | 0.95 | IGF2AS;INS-IGF2;IGF2 | Body;5'UTR;TSS1500 |
|  | cg21728792 | 0.27 | 0.038 | 0.95 | MIR483;INS-IGF2;IGF2 | TSS1500;Body |
|  | cg05203776 | 0.26 | 0.046 | 0.95 | IGF2AS;INS-IGF2;IGF2 | Body;5'UTR;TSS1500 |
|  | cg22225943 | 0.26 | 0.053 | 0.95 | IGF2AS;INS-IGF2;IGF2 | Body; 5'UTR; TSS200 |
| Cpeptide | cg17434309 | 0.28 | 0.032 | 0.98 | INS-IGF2;IGF2AS;IGF2 | Body;TSS1500; 5'UTR |
| Proinsulin | cg17434309 | 0.31 | 0.019 | 0.98 | INS-IGF2;IGF2AS;IGF2 | Body;TSS1500;5'UTR; |
|  | cg13670288 | -0.26 | 0.049 | 0.98 | INS-IGF2 | Body |
| IGF-II | cg13928782 | -0.35 | 0.007 | 0.54 | IGF2AS;INS-IGF2;IGF2 | Body; 5'UTR; TSS200 |
|  | cg21574853 | -0.34 | 0.010 | 0.54 | INS-IGF2;INS | TSS200 |
|  | cg07096953 | -0.31 | 0.018 | 0.54 | INS-IGF2;IGF2 | Body |
|  | cg20088847 | -0.30 | 0.024 | 0.54 | IGF2AS;INS-IGF2;IGF2 | Body; 5'UTR;TSS1500 |
|  | cg23889607 | 0.30 | 0.024 | 0.54 | INS-IGF2;IGF2 | Body;3'UTR; |
| HMW | cg16126291 | -0.42 | 0.0009 | **0.011** | ADIPOQ | TSS200 |
| adiponectin | cg18537894 | 0.36 | 0.006 | **0.036** | ADIPOQ | TSS1500 |
|  | cg02235049 | -0.32 | 0.016 | 0.063 | ADIPOQ | TSS1500 |
| Total | cg18537894 | 0.40 | 0.002 | **0.013** | ADIPOQ | TSS1500 |
| adiponectin | cg10681525 | -0.40 | 0.002 | **0.013** | ADIPOQ | Body |
|  | cg16126291 | -0.35 | 0.008 | **0.031** | ADIPOQ | TSS200 |
|  | cg02235049 | -0.32 | 0.016 | **0.047** | ADIPOQ | TSS1500 |

*P values of partial correlation coefficient adjusting for gestational age; P values in bold, P<0.05 at FDR<5%.

^#^ Benjamini-Hochberg corrected P values accounting for multiple tests.
